# Estimating the age of *Calliphora vicina* eggs (Diptera: Calliphoridae): determination of embryonic morphological landmarks and preservation of egg samples

**DOI:** 10.1007/s00414-015-1308-x

**Published:** 2016-01-11

**Authors:** Daniel Martín-Vega, Martin J. R. Hall

**Affiliations:** Natural History Museum, Cromwell Road, SW7 5BD London, UK

**Keywords:** Age estimation, Embryonic development, Forensic entomology, Morphology, Post-mortem interval

## Abstract

Blow fly eggs may sometimes be the only entomological evidence recovered in a forensic case, especially in cooler weather when hatching might take several days: hence, a method for estimating their age is greatly needed. However, developmental data on blow fly eggs are mainly limited to records of the time to larval hatching. The current paper describes the morphological changes occurring during embryogenesis of the blow fly *Calliphora vicina* Robineau-Desvoidy and their timing in relation to temperature, in order to determine those characters which can be used for simple egg age estimation using light microscopy. At 7.3 and 25 °C, 15 easily visualised morphological landmarks were determined in *C. vicina* living embryos, allowing for their age estimation with a resolution of 10–20 % of total egg developmental time. The observed age intervals were compared to the embryonic stages described for the fruit fly *Drosophila melanogaster* Meigen, which are used as reference data in multiple developmental studies. Moreover, current guidelines for preservation of egg samples, which recommend the placement of living eggs directly into 80 % ethanol, were tested against the hot water killing (HWK) method prior to preservation in 80 % ethanol, recommended for larval and pupal specimens. Direct placement of eggs into 80 % ethanol caused marked decomposition of samples, and no morphological landmarks were discernible. On the other hand, HWK fixation prior to preservation in 80 % ethanol enabled visualisation of 11 of the 15 age-specific morphological landmarks that were discernible in living embryos. Therefore, HWK fixation prior to preservation in 80 % ethanol is recommended for egg samples, thus unifying the protocols for collecting entomological evidence.

## Introduction

Throughout its wide geographical distribution, the blow fly *Calliphora vicina* Robineau-Desvoidy (Diptera: Calliphoridae) is often the first insect colonising cadavers, both indoors and outdoors, frequently promptly after death [1]. Colonisation starts when a female lays her eggs on the cadaver; the offspring then pass through three developmental stages (egg, larva and pupa) before the adult emerges. As the developmental rates of those immature stages are strongly influenced by temperature, a minimum post-mortem interval (PMI_min_) can be accurately estimated on the basis of forensic scene temperature information and available development data for the pertinent insect species and developmental stage [2]. Quantitative measures of age (e.g. body length) can be modelled in relation to time for *C. vicina* larvae [3], whereas different approaches have been recently developed for the pupal stage (e.g. [4–6]). However, available development data on blow fly eggs are almost entirely limited to records of the time to larval hatching [7–13], thus restricting the temporal resolution of the egg stage. The egg is by far the shortest developmental stage in blow flies, lasting about 5 % of the total duration of development [7, 10]; hence, the first batch of eggs has usually hatched by the time a cadaver is discovered. Nonetheless, blow fly eggs can sometimes be the only available entomological evidence, especially in cooler weather when hatching might take several days and when, therefore, reliable methods for estimating their age become crucial. The use of gene expression profiles is a promising and potential tool for this aim, but it requires further development, considerable expertise and specific fixation and preservation of egg samples [14]. A simple and reliable method for aging blow fly eggs is therefore essential.

A potential approach for estimating the age of blow fly eggs is the use of embryonic morphological landmarks. Developmental studies on the fruit fly *Drosophila melanogaster* Meigen (Diptera: Drosophilidae) have shown a predictable sequence of morphological changes occurring during embryogenesis [15–18]. Morphological markers have also been used for staging embryos of some Diptera species used in pest control [19, 20]. Hence, if those morphological landmarks and their timing are determined in the embryonic development of *C. vicina*, they could be used as a qualitative measure of the eggs’ age. However, published studies on blow fly embryogenesis focus mainly either on particular stages of the embryonic development [21, 22] or on the embryology of particular organs and structures [23–25]. The most comprehensive study to date is that of Starre-van der Molen [26] but, while it describes the internal changes occurring at a cellular level in detail, the external features are only briefly summarized. Moreover, Starre-van der Molen [26] reared *C. vicina* eggs at room temperature (approximately 22 °C), so no accurate data on the timing of the embryonic stages in relation to temperatures can be extracted from those observations.

Furthermore, it is always important to highlight that the analysis of entomological evidence in forensic investigations requires appropriate preserved samples in order to avoid their degradation and other post-mortem changes. In the case of larval specimens, the protocol developed by the European Association for Forensic Entomology (EAFE) recommends the killing and fixation by immersion in hot water (hot water killing or HWK) prior to storage in 80 % ethanol [27], as this method minimizes post-mortem changes in larval length and avoids the marked decomposition of tissues caused by direct placement in ethanol [28]. HWK fixation prior to storage in 80 % ethanol has been shown to be also the best preservation method for pupal specimens [29]. However, for egg specimens, the EAFE protocol [27] recommends killing and preservation by placing the live samples directly into 70–95 % ethanol. Blow fly eggs are protected by the chorion, a resistant and semi-opaque shell which must be removed for effectively visualising the embryo [16–18]. Besides being highly resistant, the chorion shows two dorsal hatching pleats holding a continuous film of air, enabling respiration when the egg is immersed in liquid [30], so the embryo might also continue its development during a certain period of time when placed alive into ethanol. Therefore, a revision of the EAFE guidelines for preservation of egg samples [27] seems necessary.

The current paper describes the morphological changes occurring during the embryonic development of *C. vicina* and their timing, in order to determine those morphological landmarks which can be easily visualised and used for estimating the age of blow fly eggs. Moreover, the quality of preservation of eggs placed directly in 80 % ethanol and eggs HWK fixed prior to storage in 80 % ethanol is tested with the aim of providing a suitable protocol for collecting and preserving egg samples recovered during forensic investigations.

## Methods

A laboratory colony of *C. vicina* was established from adults collected using a modified Redtop® fly trap (Miller Methods, Pretoria) in the Wildlife Garden of the Natural History Museum, London. Newly emerged adults from the colonies were provided with sugar, milk powder and water ad libitum during 1 week, and then also with 2 ml of pig blood (from pig liver) once daily during the following 2 weeks. The flies were then starved for 4–5 days, in order to permit adequate time for egg development and to facilitate predictable egg-laying [31]. After this, fresh pig liver was provided as an oviposition medium for 30 min, and then it was removed and a new piece of fresh pig liver was placed for another 30 min. Only this second batch of eggs was used in the experiment to minimize the occurrence of precocious eggs, which are occasionally matured within the female and laid as larvae [31].

Eggs were collected with a fine brush within 30 min of oviposition, placed on moistened tissue paper on a Petri dish, and then into an incubator under a constant temperature. The paper was checked at each egg collection and kept moistened by adding water drops; humidity levels in the Petri dish were not measured. Two constant temperatures (temperature ± standard deviation: 7.3±0.021 °C and 25±0.078 °C) were used in this experiment; 7.3 °C was chosen as a cool temperature under which the embryonic development lasts for several days, while 25 °C was used for comparison with previous studies on the embryonic development of *D. melanogaster* [15–18]. Based on data from an unpublished study carried out at the Natural History Museum (Richards et al., unpublished data), the time required from oviposition to hatching by *C. vicina* eggs is approximately 120 h at 7.3 °C and approximately 16 h at 25 °C. Accordingly, 10 eggs were randomly collected at each of the 11 10 % time intervals (i.e. from 0 % corresponding to oviposition to 100 % corresponding to hatching), placed in a 1:1 solution of sodium hypochlorite for 5 min, and then rinsed in distilled water for 2–3 min. This method removes the opaque chorion and enables the visualisation of the living embryo through the transparent vitelline membrane [16]. The dechorionated eggs were then placed on a cavity slide in Hoyer’s medium and observed and photographed with transmitted light during the following 30 min under a Leica® M165 stereo microscope. The experimental set-up was replicated three times for each temperature, using a different incubator each time to avoid potential instrument bias. Resulting images were imported into Adobe Photoshop CS4 (Adobe Systems, Inc.) and adjusted for brightness and contrast in order to improve the display of some internal structures. Terminology follows Campos-Ortega and Hartenstein [17], which includes full definitions for every term. Accumulated degree hours (ADH) required from oviposition to each time interval were calculated for both experimental temperatures using a lower developmental threshold of 1 °C, as determined for *C. vicina* in London, UK [3].

To test the killing and preservation method, 20 eggs were randomly collected at each 10 % time interval during the 7.3 °C experimental set-up; the procedure being replicated three times. Ten of those eggs were placed directly into 80 % ethanol and stored at 4 °C, while the other ten eggs were killed by immersion in near-boiling water for 30 s, transferred to 80 % ethanol and stored at 4 °C for 7–11 days. After storage, the eggs were dechorionated and visualised following the aforementioned method, and compared with the correspondent living embryos at each time interval.

## Results

### Embryonic morphological landmarks

**Fig. 1:**
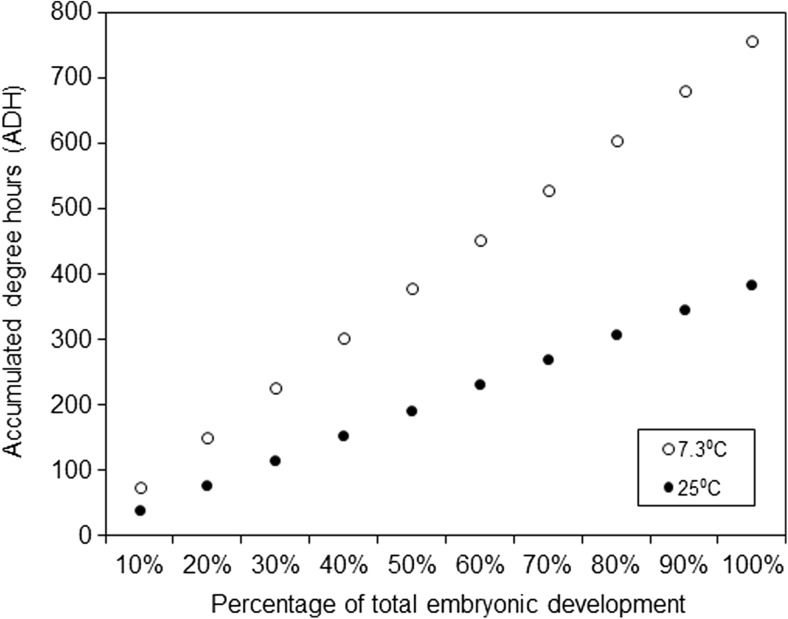
Accumulated degree hours (ADH) required from oviposition to each time interval at both experimental temperatures. ADH have been calculated using a lower developmental threshold of 1 °C, as determined for *C. vicina* in London, UK [3]

Fifteen morphological landmarks easily visualised under the stereo microscope were identified in living embryos of *C. vicina*, allowing for their age estimation with a resolution of 10–20 % of the egg development interval. The chronology of these morphological landmarks is shown in Table 1, including the ADH required from oviposition to each time interval for both experimental temperatures. The difference in the ADH requirement between both experimental temperatures increased linearly throughout embryogenesis (Fig. 1). The main morphological changes occurring at each 10 % time interval are described below. The correspondence of these intervals to the embryonic stages described for *D. melanogaster* by Campos-Ortega and Hartenstein [17] is highlighted, as their study includes thorough descriptions of the embryonic events based on histological material and the nomenclature of those stages is typically used in other developmental studies [e.g. 32]. It must be emphasised that the embryonic stages described by Campos-Ortega and Hartenstein [17] are morphological stages, each of them lasting for different time periods.

**Table 1:** Chronology of the development of the morphological landmarks diagnostic for the eleven 10 % time intervals of *Calliphora vicina* embryogenesis, visible on living embryos

Morphological landmark		0 %	10 %	20 %	30 %	40 %	50 %	60 %	70 %	80 %	90 %	100 %	Character visible in HWK + 80 % ethanol preservative
	7.3 °C	0 h	12 h (75.6 ADH)	24 h (151.2 ADH)	36 h (226.8 ADH)	48 h (302.4 ADH)	60 h (378 ADH)	72 h (453.6 ADH)	84 h (529.2 ADH)	96 h (604.8 ADH)	108 h (680.4 ADH)	120 h (756 ADH)	
	25 °C	0 h	1.6 h (38.4 ADH)	3.2 h (76.8 ADH)	4.8 h (115.2 ADH)	6.4 h (153.6 ADH)	8 h (192 ADH)	9.6 h (230.4 ADH)	11.2 h (268.8 ADH)	12.8 h (307.2 ADH)	14.4 h (345.6 ADH)	16 h (384 ADH)	
Homogeneous yolk mass (Fig. 21a)	7.3 °C	++											No
	25 °C	++											
Clear gaps (Fig. 21b)	7.3 °C	++	+++										No
	25 °C	++	+++										
Bright peripheral ring (Figs. 21c, 21d, 54c)	7.3 °C			+									Yes
	25 °C			+++									
Cephalic furrow (Figs. 21e–h, 54d, 54e)	7.3 °C			+++	+++								Yes
	25 °C			+	+++								
Dorsal folds (Figs. 21f, 21 g)	7.3 °C				++								No
	25 °C				+++								
Stomodeal invagination (Fig. 21i, 54f)	7.3 °C				+	+++	+++	+++	+++				Yes
	25 °C					+++	+++	+++	+++				
Thoracic segmentation (Figs. 32a, 54 g)	7.3 °C					+	+++	+++	+++	+++	+++	+++	Yes
	25 °C					+	+++	+++	+++	+++	+++	+++	
Clypeolabrum (Figs. 32a, 32b, 54 g, 54 h)	7.3 °C						+++	+++	+++				Yes
	25 °C						+++	+++	+++	+			
Abdominal segmentation (Figs. 32b, 54 h)	7.3 °C						++	+++	+++	+++	+++	+++	Yes
	25 °C						+	+++	+++	+++	+++	+++	
Sack-shaped gut (Figs. 32c, 54i)	7.3 °C								+++				Yes
	25 °C								+++				
Coil-shaped gut (Figs. 32d, 54j)	7.3 °C								+	+++	+++	+++	Yes
	25 °C									+++	+++	+++	
Spine bands (Figs. 54k, 54l)	7.3 °C										+++	+++	Yes
	25 °C										+++	+++	
Posterior spiracles (Figs. 32e, 32f, 54k, 54l)	7.3 °C										+++	+++	Yes
	25 °C										+++	+++	
Cephalopharyngeal skeleton (Figs. 32g, 54l)	7.3 °C											+++	Yes
	25 °C										+	+++	
Fully developed tracheal system (Fig. 32g)	7.3 °C											+++	No
	25 °C											+++	

+, character visible in <25 % of the examined individuals; ++, character visible in 25–75 % of the examined individuals; +++, character visible in >75 % of the examined individuals. Accumulated degree hours (ADH) have been calculated using a lower developmental threshold of 1 °C, as determined for *C. vicina* in London, UK [3]

#### 0 % (newly laid eggs)

**Fig. 2:**
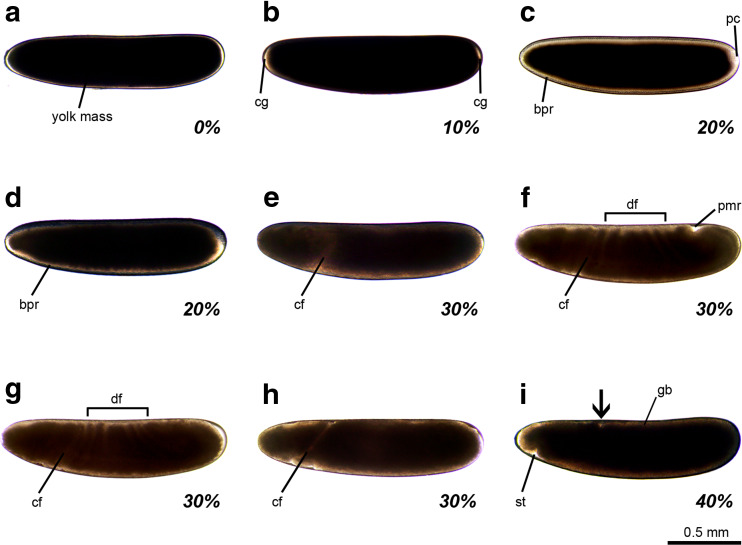
Embryonic development of *Calliphora vicina* Robineau-Desvoidy at 10 % temporal resolution, 0–40 % time intervals. The corresponding time interval is indicated under each specimen. All the specimens are laterally oriented (anterior part to the left) unless otherwise indicated (**h**). **a** 0 % time interval, **b** 10 % time interval, **c** 20 % time interval, showing the ‘bright peripheral ring’ formed by individual cells, **d** 20 % time interval, specimen showing an early gastrulation but the ‘bright peripheral ring’ is still discernible, **e** 20 % time interval, specimen showing an early gastrulation with incomplete formation of the cephalic furrow, **f** 30 % time interval, specimen showing the posterodorsal invagination of the posterior midgut rudiment, **g** 30 % time interval, specimen in which the posterodorsal invagination of the posterior midgut rudiment has already disappeared, **h** 30 % time interval, specimen in ventrolateral view showing the cephalic furrow and **i** 40 % time interval; the *arrow* indicates the dorsal indentation beside the tip of the extended germ band. The germ band can be seen as a brighter layer extending over ventral and dorsal sides. *bpr* ‘bright peripheral ring’, *cf* cephalic furrow, *cg* clear gaps, *df* dorsal folds, *gb* germ band, *pc* polar cells, *pmr* invagination of the posterior midgut rudiment, *st* stomodeal invagination

When oviposited, the egg has just been fertilized, so the zygote fills up the egg giving a uniformly coloured appearance (although bright at the periphery, often brightest at the poles) as the granular yolk mass is homogeneously distributed (Fig. 2a). This homogeneous yolk mass is observable only for a few minutes until the start of cleavage divisions. Indeed, more than 25 % of the specimens showed already the characteristic clear gaps which are diagnostic for the first 10 % interval of embryonic development (Table 1). The 0 % interval corresponds to stage 1 of Campos-Ortega and Hartenstein [17].

#### 10 % of development

Cleavage divisions take place during this interval of development. There is a contraction of the embryo which leaves two characteristic empty spaces or clear gaps at both anterior and posterior poles (Fig. 2b). The 10 % interval corresponds to stage 2 of Campos-Ortega and Hartenstein [17].

#### 20 % of development

The nuclei resultant from the cleavage divisions migrate to the periphery of the embryo and cell membranes extend inwards, so a ‘bright peripheral ring’ of individual cells is visible around the surface of the embryo (Fig. 2c). The anterior empty space disappears, whereas the posterior space is occupied by the pole cells. Most specimens from the 25 °C set-up showed this characteristic bright peripheral ring (Table 1), but a relatively low percentage of individuals (<25 %) showed already an early gastrulation characterised by an irregular surface and bright peripheral ring partially visible (Fig. 2d); in even fewer cases (<7 %), the bright peripheral ring had completely disappeared, with the cephalic furrow typical of the next development interval partially formed (Fig. 2e). Interestingly, the opposite pattern was found in the 7.3 °C set-up (Table 1): most specimens showed already an early gastrulation (Fig. 2e), whereas few still showed the bright peripheral ring (Fig. 2c). The 20 % interval corresponds to stages 3 to part of 5 of Campos-Ortega and Hartenstein [17], also including part of stage 6 in those individuals showing an early gastrulation.

#### 30 % of development

Gastrulation takes place, and the embryo shows dramatic morphological changes due to endodermal and mesodermal invaginations which form 5–7 divergent dorsal folds (Fig. 2f, g). The formation of the cephalic furrow (Fig. 2f–h) from shortening of lateral cells is also characteristic of this development interval. Dorsal folds and the cephalic furrow were observed in every specimen from this collection interval (Table 1). A clear posterodorsal invagination of the posterior midgut rudiment (Fig. 2f) can be observed in some specimens, but this invagination moves forward and folds into the embryo, disappearing rapidly (Fig. 2g). The 30 % interval corresponds to stages 6 to 9 of Campos-Ortega and Hartenstein [17].

#### 40 % of development

This developmental interval is characterised by the opening of the stomodeum (i.e. the primordia of the foregut) as a clear, anteroventral invagination of the head segment (Fig. 2i). The germ band, defined as the metameric region of the embryo [17], fully extends around the embryo; an indentation can be clearly observed beside the dorsal tip of the germ band in some specimens (Fig. 2i). Thoracic segmentation is discernible on the ventral surface of some specimens (Table 1). The 40 % interval corresponds to stages 10 and 11 of Campos-Ortega and Hartenstein [17].

#### 50 % of development

**Fig. 3:**
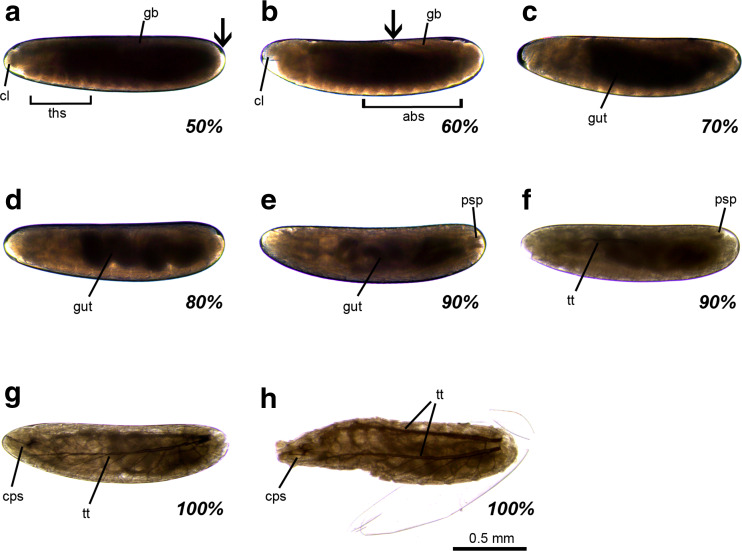
Embryonic development of *Calliphora vicina* Robineau-Desvoidy at 10 % temporal resolution, 50–100 % time intervals. The corresponding time interval is indicated under each specimen. All the specimens are laterally oriented (anterior part to the left) unless otherwise indicated (**e**, **h**). **a** 50 % time interval; the *arrow* indicates the posterior gap left by the contraction of the embryo. The germ band is usually obscured and hardly discernible during this interval. **b** 60 % time interval; the *arrow* indicates the dorsal tip of the shortened germ band. **c** 70 % time interval, specimen showing ‘sack-shaped’ gut. **d** 80 % time interval, specimen showing ‘coil-shaped gut’. **e** 90 % time interval, specimen in dorsal view. **f** 90 % time interval, specimen with tracheal trunks partially visible. **g** 100 % time interval. **h** 100 % time interval, first instar larva in dorsal view hatching from the vitelline membrane. *abs* abdominal segmentation, *cl* clypeolabrum, *cps* cephalopharyngeal skeleton, *gb* germ band, *psp* posterior spiracles, *ths* thoracic segmentation, *tt* tracheal trunk

The stomodeum invaginates further and the clypeolabrum (i.e. the fused plate of the clypeus and labrum) can be observed as a clear, anterior protuberance (Fig. 3a). The germ band starts to shorten during this interval and the posterior part of the embryo retracts from the vitelline membrane, leaving a clear gap which is, however, short lived, disappearing by the 60–70 % developmental stage (Fig. 3a). Thoracic segments become distinct on the ventral surface of the embryo; they were observed in every specimen from this and subsequent collection intervals (Table 1). Abdominal segments were also discernible on the ventral surface of a few specimens (Table 1). The 50 % interval corresponds to stage 12 of Campos-Ortega and Hartenstein [17].

#### 60 % of development

As the involution of the head segment begins, a dorsal fold starts to progressively cover the clypeolabrum, although it is still clearly distinct (Fig. 3b). The germ band shortens on the dorsal surface and the posterior gap between the embryo and the vitelline membrane almost completely disappears (Fig. 3b). Abdominal segments become evident on the ventral surface of the embryo. The 60 % interval corresponds to stage 13 of Campos-Ortega and Hartenstein [17].

#### 70 % of development

The dorsal fold slides over the clypeolabrum and covers it completely (Fig. 3c). In the central region, the gut can be visualised as a closed, ‘sack-shaped’ tube containing the yolk (Fig. 3c). The 70 % interval corresponds to stages 14 and 15 of Campos-Ortega and Hartenstein [17].

#### 80 % of development

The gut becomes ‘coil-shaped’ as the yolk is gradually digested (Fig. 3d). The gut will be observed until the end of the embryonic development (Table 1), becoming thinner during subsequent development intervals (Fig. 3e). The embryo starts to move actively during this interval. The 80 % interval corresponds to part of stage 16 of Campos-Ortega and Hartenstein [17].

#### 90 % of development

Secretion of the cuticle starts at the epidermis and at the tracheal system, so the spine bands and the paired posterior spiracles are discernible (Fig. 3e). However, peristaltic contractions of the gut can be observed and the active movements of the embryo increase significantly, making it difficult to image the spine bands. As the tracheal trunks are progressively filled with air, they can be partially visible in some specimens (Fig. 3f). The sclerotized cephalopharyngeal skeleton can also be discernible in some specimens (Table 1). The 90 % interval corresponds to part of stage 16 of Campos-Ortega and Hartenstein [17].

#### 100 % of development

The paired tracheal trunks are progressively filled with air and the complete tracheal system is visible (Fig. 3g). The cephalopharyngeal skeleton also becomes clearly distinct. The embryo becomes the first instar larva, which actively tears the vitelline membrane with the tips of the mouth hooks in order to hatch (Fig. 3h). The 100 % interval corresponds to stage 17 of Campos-Ortega and Hartenstein [17].

### Preservation of egg samples

**Fig. 4:**
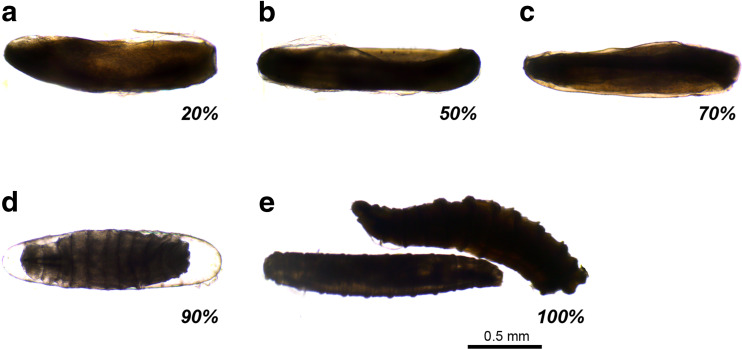
Eggs of *Calliphora vicina* Robineau-Desvoidy collected at 10 % time intervals of the total embryonic development and placed directly in 80 % ethanol. The corresponding time interval is indicated under each specimen. All the specimens are laterally oriented (anterior part to the left) unless otherwise indicated (**d**). **a** 20 % time interval, **b** 50 % time interval, **c** 70 % time interval and **d** 90 % time interval, specimen in ventral view showing the anatomy of a first instar larva. Note that the chorion has not been removed. **e** 100 % time interval, first instar larvae hatched during storage in 80 % ethanol

Preservation of eggs in 80 % ethanol without previous fixation resulted in a marked decomposition of tissues, making it impossible to visualise the morphological characters diagnostic for age on the specimens collected from 0 to 80 % time-collection intervals (Fig. 4). Moreover, after several days of storage in 80 % ethanol, the chorion became semi-transparent (Fig. 4d). On the other hand, all the specimens collected at both 90 and 100 % time intervals and placed directly in 80 % ethanol showed the morphology of the first instar larva (Fig. 4d), with some of them having hatched in ethanol (Fig. 4e). In all cases, the larvae showed signs of decomposition and discolouration (Fig. 4d, e).

**Fig. 5:**
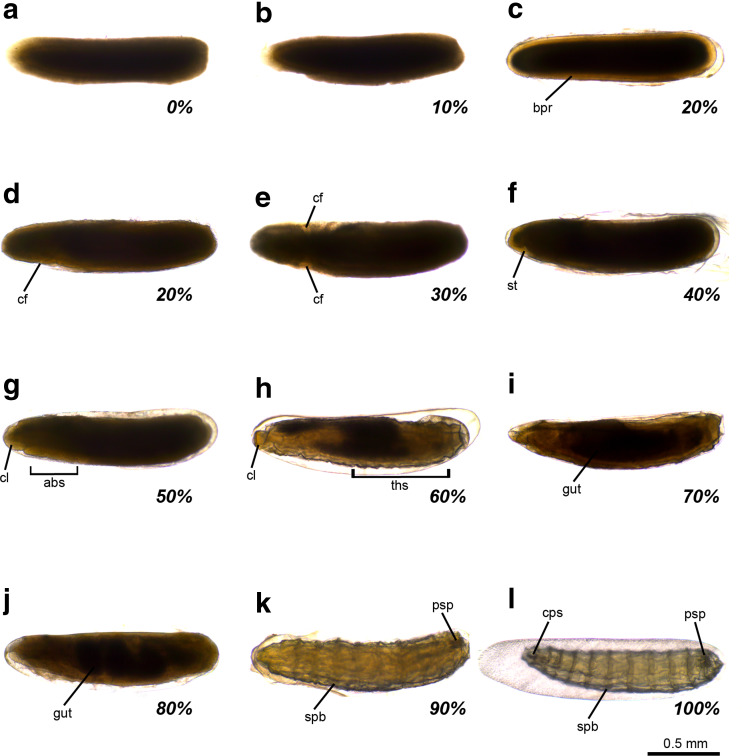
Eggs of *Calliphora vicina* Robineau-Desvoidy collected at different 10 % time intervals of the total embryonic development and preserved in 80 % ethanol for 1–2 weeks after hot water killing (HWK) fixation. The corresponding time interval is indicated under each specimen. All the specimens are laterally oriented (anterior part to the left) unless otherwise indicated (e). **a** 0 % time interval, **b** 10 % time interval, **c** 20 % time interval, showing the ‘bright peripheral ring’ formed by individual cells, **d** 20 % time interval, specimen showing an early gastrulation with incomplete formation of the cephalic furrow, **e** 30 % time interval, specimen in ventral view showing the cephalic furrow, **f** 40 % time interval, **g** 50 % time interval and **h** 60 % time interval. Note that the chorion has not been removed. **i** 70 % time interval, specimen showing ‘sack-shaped’ gut, **j** 80 % time interval, specimen showing ‘coil-shaped gut’, **k** 90 % time interval and **l** 100 % time interval. Note that the chorion has not been removed. *abs* abdominal segmentation, *bpr* ‘bright peripheral ring’, *cf* cephalic furrow, *cl* clypeolabrum, *cps* cephalopharyngeal skeleton, *psp* posterior spiracles, *spb* spine bands, *st* stomodeal invagination, *ths* thoracic segmentation

On the other hand, HWK fixation prior to storage in 80 % ethanol allowed for the visualisation of 11 of the 15 morphological landmarks on preserved eggs (Fig. 5; Table 1), although with less resolution than the living embryos (Figs. 2, 3). As mentioned, the chorion became semi-transparent after several days of storage in 80 % ethanol, sometimes making it possible to identify the morphological landmarks without removing it (Fig. 5h, l). Collapse of the vitelline membrane prevented the visualisation of the clear gaps typical of the 10 % developmental interval (Fig. 5b, compare with Fig. 2b), as well as the correct visualisation of the dorsal folds characteristic of the 30 % development interval (compare Fig. 5e with Fig. 2f, g), although the cephalic furrow could be observed (Fig. 5e). Moreover, due to protein coagulation by HWK fixation, the tracheal system is not discernible in the 100 % developmental interval (Table 1), while other morphological landmarks (i.e. the posterior spiracles and the cephalopharyngeal skeleton) were sometimes barely discernable (Fig. 5l).

## Discussion

### Embryonic morphological landmarks

Previous studies on the egg development of forensically important blow flies suggested that the only external age-diagnostic markers in non-dechorionated eggs become visible in the very last hours of the embryonic development, when distinct mouthparts and spine bands can be visualised as the first instar larva is almost ready to hatch [10, 12]. Particularly, Sanit et al. [12] only observed clear spine bands from the 80 to 90 % period of total embryonic development in the blow fly *Hypopygiopsis tumrasvini* Kurahashi, with no obvious features at earlier stages. This severely limited the temporal resolution of an egg aging method based on the identification of morphological landmarks. However, visualisation of the diagnostic characters can be solved with dechorionation of eggs, as it reveals the morphology of the embryo, making it possible to identify the morphological changes occurring during egg development as already applied to other Diptera species [16, 17, 19, 20]. Dechorionation of eggs is a very simple method, not requiring special equipment or particular expertise. Although staining methods can enhance the contrast between cell layers and yolk, thus enhancing the visualisation of some internal morphological characters [18], a simple and fast mounting of the embryos using Hoyer’s medium or a solution of sodium chloride [16] is suitable for visualising and identifying the age-specific morphological landmarks determined here (Table 1). It must be noted that the variation in the extent of the germ band during embryonic development (Figs. 2i, 3b) could be an additional informative marker (see Campos-Ortega and Hartenstein [17] for further information on this structure). Nevertheless, it has not been included in the current table of diagnostic landmarks (Table 1) because it is not always easily discernible, becoming only prominent during shortening (Fig. 3b), probably due to an increasing cell density [17]. Before shortening, the maximum extent of the germ band can be identified by a dorsal indentation (Fig. 2i), but it must be emphasised that this is a variable feature and frequently obscured by the folds of the serous membrane [17].

Overall, the morphological characters described and their chronology are consistent with those described for other Diptera species [15–20, 32], including the previous study on *C. vicina* embryogenesis [26]. Nevertheless, it must be highlighted that the 5–7 divergent dorsal folds observed during gastrulation at 30 % of embryonic development of *C. vicina* (Fig. 2f, g) are more numerous than the three folds observed in *D. melanogaster* [17], being more similar to the up to six folds observed in the more closely related blow fly *Lucilia sericata* (Meigen) [32]. Mellethin et al. [32] suggested that such higher number of dorsal folds formed during gastrulation might be due to the different dimensions of the body axes, with a much greater length-to-thickness ratio in blow fly eggs than in *Drosophila* eggs.

Regarding the timing of the developmental intervals, it must be noted that previous descriptions of the embryonic stages included only approximations of the duration of each stage and highlighted the difficulty of delimiting the embryonic stages [17]. Indeed, when applying the current age-specific morphological landmarks (Table 1), it should be taken into account that embryonic development is a continuous process and that the morphological events observed at the end of a development interval continue during part of the following interval. In any case, the time intervals described here are consistent with the times suggested for *C. vicina* embryogenesis by Starre-van der Molen [26]. Interestingly though, when compared to the times suggested for *D. melanogaster* at 25 °C [17], the first stages of embryonic development appear to progress slightly faster in *C. vicina* (Table 1). Mellenthin et al. [32] also suggested shorter stages in the first part of the embryonic development of *L. sericata*. Different duration of the embryonic stages has been observed in closely related species of the genus *Drosophila* [20], so the development intervals determined in the current study for *C. vicina* might not be valid for other Calliphoridae species.

It is worth mentioning the difference observed at the 20 % development interval between the two temperature set-ups (Table 1), suggesting a proportionately slightly shorter development during the first stages of embryogenesis at lower temperatures. Lower tolerance of young embryos to cold has been observed in *L. sericata* [33] and *Drosophila suzukii* (Matsumura) [20], so shortening of earlier stages at lower temperatures might be an adaptive strategy of cold-tolerant species like *C. vicina*. However, our current data do not allow us to draw further conclusions on this issue and, to the best of our knowledge, there are no studies on the duration of the embryonic stages at different temperatures in other blow fly species. It is also beyond the scope of the current study to discuss the difference in the ADH requirements between the two experimental temperatures (Fig. 1). However, our result concurs with other studies of *C. vicina* that report higher values of ADH at lower temperatures [3, 27, 34]. This variation requires further investigation, including use of more experimental temperatures than the two used in the present study.

### Preservation of egg samples

Direct placement of living entomological samples into ethanol has been shown to cause marked decomposition of tissues following death of the samples, frequently resulting in shrinkage and discolouration [4, 28, 29]; this is also clearly the case in blow fly eggs as indicated by the current results (Fig. 4). Moreover, as ethanol does not instantly kill the specimens, it might lead to erroneous PMI_min_ estimations, particularly in the last hours of egg development, when first instar larvae may hatch in the preservative (Fig. 4). For these reasons, direct placement of live eggs into ethanol should be avoided when collecting egg samples at the forensic scene.

As mentioned, HWK fixation prior to preservation in 80 % ethanol is already recommended for larval and pupal samples collected at forensic scenes [27, 29]. This fixation and preservation method also allows for the visualisation of most age-diagnostic morphological landmarks in egg samples (Fig. 5; Table 1); hence, it is also recommended for eggs. Dechorionation may not be strictly needed in some cases as the chorion usually becomes semi-transparent after several days of storage and the fixed embryo can be visualised inside (Figs. 5h, l). However, to achieve the best possible resolution, it is preferable in most cases to remove the chorion.

Finally, as a guideline for forensic practice, the egg samples collected at a forensic scene should ideally be divided into two batches: one should be placed on moistened tissue paper in vials, stored in a cool bag with a temperature data logger and transferred to an expert for rearing as soon as possible, and the other one should be HWK fixed (as soon as possible after collection, recording the time) prior to storage in 80 % ethanol for a subsequent morphological analysis at the laboratory. This recommendation of fixation and storage method unifies the protocol for collecting entomological evidence as already recommended for both larval and pupal samples [27, 29].
